# The influence of teachers’ psychological capital on the quality of work life: exploring the mediating impact of emotions

**DOI:** 10.3389/fpsyg.2025.1557030

**Published:** 2025-02-25

**Authors:** Hale Erden

**Affiliations:** Educational Sciences Department, Faculty of Educational Sciences, Final International University, Kyrenia, Cyprus

**Keywords:** psychological capital, emotions, quality of work life, mediating impact, teachers

## Abstract

**Introduction:**

The aim of this study is to investigate whether emotions play a mediating role in determining the effect of teachers’ psychological capital (PsyCap) on the quality of work life (QWL).

**Methods:**

The current study presents an investigation on the mediating role of emotions and their effect on teachers’ PsyCap at their work life. The study used scales including Psychological Capital Scale (PsyCapS), Quality of Work Life Scale (QWLS) and Emotions Scale (ES). The scales were all applied to administrators and teachers in state schools in TRNC. The sample of the study consists of 369 teachers and school administrators. Data were analyzed using SPSS 27.0 for descriptive statistics and correlation analyses. In addition, structural equation modeling was performed with AMOS to examine the potential mediating effect of emotions on the relationship between PsyCap and QWL.

**Results:**

As a result, the mediating role of emotions in the relationship between teachers’ PsyCap and perceptions of QWL was fully supported. There was a significant correlation between teachers’ PsyCap, perceptions of QWL and emotions. PsyCap was found to have a significant and positive effect on both emotions and perceptions of QWL. In addition, PsyCap was found to have a significant positive relationship with QWL.

**Discussion:**

Among TRNC teachers, PsyCap has a significant positive relationship with both emotions and perceptions of QWL. PsyCap can directly or indirectly increase the perception of QWL through the relationship with emotions. Therefore, teachers’ positive PsyCap can positively increase their emotions and perceptions of QWL.

## 1 Introduction

Emotions play a crucial role in our daily lives. Goleman (1995) notes that they provide meaning to our experiences and guide our behaviors both personally and professionally. American Psychological Association (2021) defines emotion as “a complex response pattern that includes experiential, behavioral, and physiological elements.” Lawler and Thye (1999, p. 219) describe emotions as brief evaluative states-either positive or negative-with physiological, neurological, and cognitive aspects. Understanding emotions is vital in organizational behavior, as they influence actions in various ways (Akçay and Çoruk, 2012). Emotions motivate us, prompting behaviors that address the immediate concerns that triggered them (Frijda, 1986).

The school has an organizational structure centered on people who come from and return to society (Bursalıoğlu, 2011). It features multidimensional interactions among various groups, including administrators, teachers, students, and parents, as well as external sources. This leads to intense human relations and emotions within the school (Barutçugil, 2002; Oplatka and Arar, 2018; Yamamoto et al., 2013). Hargreaves (1998) emphasized the significance of teachers’ emotional dimensions, and recent research on teacher emotions has notably increased (Šarić, 2015; Burić et al., 2017; Frenzel et al., 2016).

Emotion is a powerful force that shapes our thinking, decision-making, actions, social relationships, wellbeing, and overall physical and mental health (Izard, 2010). Güngör and Titrek (2024) have demonstrated that school principals’ emotional management skills are effective in the decision-making process. Within the educational realm, teachers are aptly designated as emotion workers (Yin et al., 2019). Recognizing the significance of teachers’ emotions is essential, as they profoundly influence professional development and the quality of teaching (Lavy and Eshet, 2018). Various factors affect teachers’ emotional states, encompassing personal characteristics, evaluative processes, and broader social, cultural, and political contexts (Fried et al., 2015). Furthermore, Lai et al. (2024) argue that the elements influencing teachers’ emotions can be streamlined into three overarching levels: the personal qualities of teachers, the contextual factors of their work environment, and the surrounding sociocultural influences. By understanding these dynamics, the teaching profession can be enhanced, ultimately improving educational outcomes for students.

Teachers bring a multitude of personal qualities to their roles, including unique characteristics, educational beliefs, professional identities, emotional intelligence, and essential competencies. The work context plays a critical role as well, shaped by the organizational climate, institutional norms, interpersonal interactions, availability of educational resources, and the physical environment. Furthermore, sociocultural factors-such as the culture surrounding curriculum reform, family education approaches, and community values-are vital to a teacher’s experience. The Theory of Emotional Events highlights that experiences in the workplace evoke emotional responses, which significantly influence attitudes and behaviors (Weiss and Cropanzano, 1996). Teachers’ emotions are intricately connected to their students and the learning process, their own identities as educators, and the relationships they cultivate with colleagues. These feelings are also influenced by support from school leaders, expectations from parents, and overarching educational policies, all contributing to the context in which teaching occurs (Wu and Chen, 2018). While external factors from within the school, community, and society can impact teachers’ feelings, the emotional responses triggered by engaging with students remain the most common and intense (Chen, 2019). Recognizing and addressing these emotional dynamics is crucial for fostering a positive educational environment.

Teachers frequently experience a wide range of discrete emotions such as joy, contentment, pride, love, anger, fatigue, hopelessness, anxiety, shame, or boredom when teaching and interacting with students (Sutton and Wheatley, 2003; Frenzel et al., 2016; Burić et al., 2017; Chen, 2019). Yıldırım (2019) determined that schools have the strongest structure in the relationship network, which includes the feelings of trust, pride, enthusiasm, courage, satisfaction, contentment, belief and peace.

Hargreaves (2005) proposed the theory of emotional geography as a framework for studying the relationship between teachers’ emotions and educational change. This theory has five dimensions: cultural, moral, professional, political and physical geography. The concept of affective geographies helps to identify supports and threats to the basic affective bonds and understandings of schooling that arise from forms of distance or closeness in people’s interactions or relationships. To ensure the effectiveness of educational changes and policies, administrators need to consider the emotional factors that influence teachers (Hargreaves, 2005).

While good working conditions can enable teachers to work comfortably and satisfactorily, poor working conditions not only affect the quality of work but can also negatively affect teachers’ mental health (Kidger et al., 2016). They also make it difficult to retain talented teachers in the system. Negative emotions are considered to be direct reactions to specific events that are related to the individual. They are especially likely to occur when events are perceived as threatening or when individuals perceive that they lack the ability to manage (Kiefer and Barclay, 2012).

Teachers’ emotional states affect many factors such as the quality of education, quality of teaching, school climate, students’ achievements, behaviors, participation in classroom activities, teachers’ classroom discipline behaviors, classroom management, teaching styles and self-efficacy beliefs (Frenzel, 2015; Frenzel et al., 2009; Hagenauer et al., 2015; Hosotani and Imai-Matsumura, 2011; Pekrun, 2014; Taxer and Frenzel, 2015). In this sense, regulating emotions is almost essential for daily functioning and wellbeing in itself (Cohen and Ochsner, 2018).

### 1.1 The effect of PsyCap on emotions

In recent years, the increasing interest in positive psychology and the increase in studies in this field have led to the emergence of concepts that include positive approaches, leaving aside negative emotions and behaviors (Klusmann et al., 2008; Ma, 2023; Sun et al., 2022; Zewude and Hercz, 2022).

PsyCap is a concept that tries to explain organizational life and employee characteristics by emphasizing the positive characteristics of the employee. Teachers with strong PsyCap are more likely to engage in effective emotion regulation strategies, which help them manage workplace challenges. Emotional regulation serves as a key mediator between PsyCap and teacher burnout, suggesting that those with greater psychological resources experience fewer negative emotional states (Liu and Du, 2024; Madigan et al., 2023). Similarly, Sun et al. (2022) highlighted that ego-resiliency, a component of PsyCap, strengthens emotional stability and fosters a sense of purpose in work. Another theoretical relationship of PsyCap is the model of expanding and building positive emotions (Fredrickson, 1998). This model suggests that positive emotions lead to higher levels of cognitive and emotional functioning (Fredrickson and Losada, 2005). Similarly, a high level of PsyCap not only reduces stress but also enhances positive emotions, such as enthusiasm and job satisfaction. Positive emotions, in turn, contribute to increased work engagement, as teachers who feel emotionally supported are more likely to be motivated and effective in their roles (Ma, 2023). This aligns with research emphasizing the role of optimism in maintaining emotional balance under pressure (Zewude and Hercz, 2021).

In this context, harnessing positive psychological resources can enhance an individual’s range of behaviors and serve as a vital shield against negative or adverse situations. Research indicates a positive and moderate correlation between positive emotions and PsyCap, while negative emotions demonstrate a negative and low correlation with it (Altunok, 2021). Moreover, employees with high PsyCap tend to experience diminished superficial and deep acting, while their natural emotional expressions flourish positively (Beğenirbaş, 2015). This highlights the critical importance of nurturing PsyCap for improved emotional wellbeing in the workplace. Overall, psychological capital has a profound effect on emotions by fostering resilience, reducing stress, and promoting positive emotional experiences. By strengthening PsyCap through training and interventions, individuals, especially those teaching and serving at the educational arena, can develop better emotional coping mechanisms, leading to improved wellbeing and job performance (Gupta et al., 2019; Luthans et al., 2006).

### 1.2 The effect of emotions on the QWL

Emotions play a critical role in shaping the QWL, influencing motivation, job satisfaction, and overall wellbeing. Positive emotions such as enthusiasm, joy, and fulfillment enhance workplace engagement, while negative emotions like stress, frustration, and burnout can lead to decreased productivity and dissatisfaction. For educators, in particular, emotions affect personal wellbeing and impact their ability to create a supportive learning environment for students (Zewude and Hercz, 2022).

Ashforth and Humphrey (1995) emphasized that emotions are intertwined with organizational life and are an inseparable part of organizational life, and therefore more importance should be given to the emotional states of employees. According to Pugh (2001), work life is an emotional experience and employees express their emotions while performing their roles. When employees experience positive emotions, they are more likely to feel motivated and engaged in their work. Research suggests that psychological capital (PsyCap), a combination of optimism, resilience, hope, and self-efficacy, helps foster these positive emotional states, leading to a better work life experience (Luthans et al., 2007b). Teachers with higher PsyCap, for example, report greater job satisfaction and lower levels of stress, which enhances their overall QWL (Ma, 2023). Furthermore, positive emotions contribute to stronger relationships with colleagues, fostering a more collaborative and supportive work environment (Liu and Du, 2024; Madigan et al., 2023).

According to Hareli, Rafaeli and Parkinson, the theory of emotional events is basically based on the theory that emotions affect behavior. They explained this as follows; the emotions an individual experiences while performing a task affect their performance while performing the next task. They also emphasized that the general emotional climate of a group affects the performance of individuals and the overall performance of the group (Hareli et al., 2008). Conversely, negative emotions can significantly hinder job performance and QWL. Chronic stress and emotional exhaustion have been linked to burnout, a common issue in demanding professions like teaching (Sun et al., 2022). Teachers who struggle with emotional regulation may find it difficult to maintain engagement, leading to reduced effectiveness in the classroom and increased job dissatisfaction (Zewude and Hercz, 2021). Studies indicate that emotional regulation plays a crucial role in mediating the relationship between psychological capital and burnout, emphasizing the importance of developing coping strategies to maintain emotional balance (Liu and Du, 2024; Madigan et al., 2023).

QWL is the general quality of an individual’s work life. Quality of life includes factors such as income, health, social relationships, as well as other factors such as happiness and satisfaction. The emotions experienced by an individual significantly affect their organizational behavior (Weiss and Cropanzano, 1996). It shows that emotional exhaustion can be managed by ensuring QWL (Permarupan et al., 2020). In organizations with a positive emotional climate, relationships between employees are more effective, which makes it difficult for negative emotions to be present. Synergy occurs within organizations and is believed to enhance performance. To improve QWL, organizations should prioritize emotional wellbeing by creating a supportive work environment, promoting stress management programs, and fostering psychological capital among employees. Encouraging the development of resilience and emotional regulation strategies can help individuals manage workplace challenges more effectively, ultimately leading to a more positive and fulfilling work experience (Monteiro and Joseph, 2023).

Emotions influence people, especially by those around their physical surroundings, and the variations in task completion. Organizational life elicits a range of emotions, and the emotional exchanges among members shape the environment. These emotional experiences impact behaviors, attitudes, and cognition. The interplay of emotions within a team creates a complex network of relationships, where collaboration can either thrive or falter based on the prevailing emotional climate. Positive emotional exchanges, such as mutual support and recognition, can motivate individuals toward shared goals, fostering a sense of belonging and commitment to the organization’s mission. Conversely, negative emotions like frustration or jealousy can create divisions, leading to disengagement and decreased productivity. Additionally, the spatial configuration of an organization plays a crucial role in shaping emotional dynamics. Open office layouts may encourage spontaneous interactions and camaraderie, while isolated workspaces can lead to feelings of disconnection and loneliness. Therefore, it is essential for leaders to create environments that promote not only physical accessibility but also emotional connectivity among team members. As organizations deal with the complexities of collective emotions, it becomes increasingly vital to embrace emotional intelligence as a cornerstone of effective leadership. Leaders who are attuned to the emotional needs of their teams can implement strategies that enhance morale and cultivate resilience in the face of challenges. By recognizing and valuing the emotional contributions of each member, organizations can harness a richer tapestry of collaboration that drives innovation and growth. Ultimately, the emotional landscape of an organization can be both a source of strength and a potential pitfall. By fostering a culture that prioritizes open communication, empathy, and genuine appreciation for one another, organizations can maximize the synergy that arises from their shared emotional experiences, unlocking significant potential for collective achievement. Emotional expressions enhance our performance and contribute to a better work environment (Elfenbein, 2023). The QWL encompasses factors such as employee satisfaction, employer attitudes, social dynamics, work-family balance, and the overall impact of work on individuals (Martel and Dupuis, 2006). Thus, it is likely that emotions influence perceptions of QWL.

### 1.3 The effect of PsyCap on the QWL

PsyCap is an entity composed of individual characteristics and qualities that support the expression of positive resources and abilities, and from this perspective, it can be considered close to the definition of eudemonic wellbeing. This structure supports people to cope effectively with daily life: acting proactively, trusting in their possibilities, and looking positively at future scenarios without being discouraged by difficulties (Santisi et al., 2020).

PsyCap consists of four basic components: resilience, optimism, hope, and self-efficacy. Resilience refers to an individual’s mental ability to quickly adapt to and recover from difficulties, failures, and setbacks. Optimism refers to an individual’s positive attitudes and attributions about real and future objective events. Hope refers to an individual’s mental state of adjusting their cognitive pathways to meet the needs of a goal. Self-efficacy refers to an individual’s sense of competence to challenge a goal and trust in success. These processes reflect to some extent how emotion regulation strategies are employed. Bertieaux et al. (2024) found that PsyCap and wellbeing, which is a dimension of QWL, are closely linked and have been studied especially in the field of educational sciences. They determined that there is a significant relationship between English teachers’ positive PsyCap and their work engagement (Xu, 2023).

Emotions significantly influence the QWL, shaping job satisfaction, engagement, and overall wellbeing. While positive emotions enhance motivation and workplace relationships, negative emotions can contribute to stress and burnout. By investing in emotional wellbeing and psychological capital, organizations can create a healthier, more productive work environment, ultimately benefiting both employees and the organization as a whole (Bakker et al., 2014; Luthans and Broad, 2022).

### 1.4 The mediating effect of emotions

Emotions are internal psychological processes that consist of multiple components, including an emotional core, cognitions, physiological arousal, expressive behavior, and action tendencies (Scherer, 1984). In the field of organizational behavior, one might question the impact of emotional events on data related to perception, attitudes, or behaviors. For instance, when evaluating the perceived support an individual experiences, could an emotional event from that day or from the past influence their perception? Numerous studies have explored the mediating role of emotions in organizational contexts. Notable examples include: the impact of stereotype threat as a determinant of burnout or work engagement (Bedyńska and Żołnierczyk-Zreda, 2015); the mediating role of positive and negative emotions in the relationship between emotional contagion and empathy fatigue (Soyumert, 2021); and the effect of exploitative leadership on counterproductive work behavior through a dissociated emotions approach (Guo et al., 2023). Additionally, there are investigations into the effect of perceived organizational justice on counterproductive work behaviors, focusing on the mediating role of negative emotions (Aisha et al., 2022), as well as studies on nurses’ emotions, emotional labor, and job satisfaction (Lee and Jang, 2020), and the mediating role of emotions in fatigue and burnout among police officers (Basinska et al., 2014). This study aims to examine the relationship between teachers’ PsyCap and their perceptions of QWL, particularly emphasizing the mediating role of perceived emotions.

The current study presents an investigation on the mediating role of emotions and their effect on teachers’ PsyCap at their work life. The hypotheses on PsyCap and its relationship with QWL and with emotions, as well as emotions and their relationships with QWL and their mediation role between PsyCap and QWL will be tested. This study offers significant contributions to the existing literature by examining the intricate relationships between teachers’ PsyCap, their emotional experiences, and the overall QWL.

Teaching is a demanding profession that often leads to stress and burnout, which can negatively impact educators’ wellbeing and performance. Therefore, it is essential to understand the factors that can enhance teachers’ QWL to develop effective support interventions. This study examines the potential mediating role of emotions in the relationship between teachers’ PsyCap and their perceived QWL.

PsyCap consists of hope, efficacy, resilience, and optimism, and has been connected to various positive organizational outcomes. Previous research has indicated that teachers’ PsyCap is a positive predictor of workplace wellbeing, with work-meaning cognition acting as a mediator in this relationship. However, the specific role of emotions in mediating the relationship between PsyCap and QWL in the education sector has not been thoroughly explored. Teachers play a critical role in shaping future generations, yet many struggles with burnout due to high workloads, emotional strain, and increasing professional demands (Agyapong et al., 2022). If emotions do mediate the PsyCap, QWL relationship, interventions aimed at enhancing emotional regulation and resilience could significantly improve teacher retention, satisfaction, and performance.

Given the myriad challenges faced by educators, it’s crucial to foster PsyCap among them. This research aims to clarify how positive psychological resources, mediated by emotional experiences, contribute to an improved QWL. It specifically seeks to identify whether positive emotions can act as a conduit through which PsyCap enhances teachers’ QWL. While previous studies have shown that PsyCap improves workplace engagement and wellbeing (Zewude and Hercz, 2022), research on how emotions function as a bridge between PsyCap and QWL in teachers is still limited (Ma, 2023).

Despite growing interest in the role of PsyCap in workplace settings, few studies have investigated how emotions specifically influence the relationship between PsyCap and QWL in the education sector. Sun et al. (2022) seek to fill that gap by providing a more nuanced understanding of how positive psychological resources contribute to teachers’ professional wellbeing. Recent studies have delved into the intricate dynamics between teachers’ PsyCap, their emotional experiences, and the overall QWL. Similarly, Ma (2023) explored how emotion regulation influences work engagement among English teachers, highlighting the mediating role of PsyCap in this relationship. At the same way, Zewude and Hercz (2021) investigated the mediation role of coping with stress between PsyCap and teacher wellbeing, providing insights into how these factors interplay in educational settings. These studies contribute to understanding of how emotions and PsyCap collectively impact teachers’ professional experiences. By leveraging concepts from positive psychology and organizational behavior, the current study enhances understanding of the psychological mechanisms involved, particularly the mediating role of emotions in the influence of PsyCap on QWL.

## 2 Methods

### 2.1 Participants

The sample of the study consists of a total of 369 teachers and school administrators working in TRNC state primary, secondary and high schools and willing to participate in the study. Simple random sampling strategy was used in the current study. 323 of the participants in the study were teachers and 46 were school administrators. In Northern part of Cyprus, to become a school administrator a teacher takes an exam, and school administrators are basically teachers. 277 (75.1%) of the total participants were female and 92 (24.9%) were male. Table 1 provides demographic information about the participants in the study as frequencies and percentages.

**Table 1:** Occupational and demographic characteristics of the study sample.

Variable	Level	*N*	Percentage
Sex	Female	277	75.1
	Male	92	24.9
Marital status	Married	277	75.1
	Single	92	24.9
Age	Below 30	38	10.3
	Between 31 and 40	133	36.0
	Between 41 and 50	143	38.8
	Above 51 and over	55	14.9
Educational status	Bachelor’s degree	200	54.2
	Master’s degree	155	42.0
	Doctorate	14	3.8
Title	Teacher	323	87.5
	School administrator	46	12.4
Seniority	Between 1 and 10	124	33.6
	Between 11 and 20	117	31.7
	Above 21 and over	128	34.7
School type	Primary school	108	29.3
	Secondary school	55	14.9
	General high school	101	27.4
	College	68	18.4
	Vocational high school	37	10.0
Years teaching at school	Between 1 and 10 years	270	73.2
	Between 11 and 20 years	75	20.3
	Above-21 and over	24	6.5

### 2.2 Research model and hypotheses

**Figure 1:**
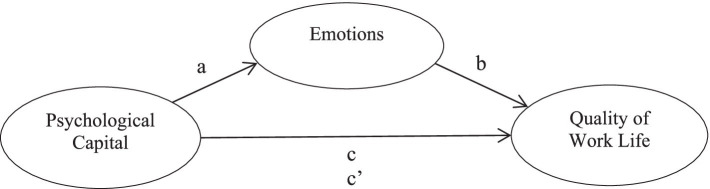
Conceptual model of the current study.

The study is a research in the relational screening model. In this study, based on the literature review mentioned above, a conceptual research model is given in Figure 1. According to this model, PsyCap is the independent variable, emotions are the mediator variable and QWL is the dependent variable. The mediator variable is defined as the variable that mediates the effect of the independent variable on the dependent variable (Saruhan and Özdemirci, 2013, p. 168). Using this model, the following hypotheses will be tested:

*H1*: PsyCap has relationships with QWL.

*H2*: PsyCap has relationships with emotions.

*H3*: Emotions have relationships with QWL.

*H4*: Emotions have mediation role between PsyCap and QWL.

The study first examined the predictive role of PsyCap on the perception of QWL using path analysis. Next, it investigated how PsyCap predicts the perception of QWL through emotions, as depicted in the model in Figure 1, using structural equation modeling.

### 2.3 Ethics statement

The data collection processes carried out within the scope of this research were approved by the Final International University Ethics Committee with the decision number 2024/13/19 dated 07.06.2024.

### 2.4 Data analysis

Data analysis was performed using SPSS 27 and AMOS 22 software programs. The SPSS 27 software package was specifically used to conduct descriptive statistics, correlation analysis, and mediation analysis. In parallel, the AMOS 22 software package was effectively employed for structural equation modeling (SEM). Confirmatory factor analysis was conducted to rigorously evaluate the fit of the scales utilized in this study. A range of fit indices, including χ^2^/df, RMSEA, TLI, NFI, GFI, CFI, and SRMR, were systematically applied to assess the models’ goodness of fit.

The construct validity of the measurement tools used in the study was tested using confirmatory factor analysis based on the data collected in this study. Bootstrapping method was used to examine the mediation effect.

The goodness of fit values in Table 2 show the cut-off points of good fit and acceptable fit values for the confirmatory factor analysis and hypothesis testing of the QWLS.

**Table 2:** Model goodness of fit.

Fit measure	Good fit	Acceptable fit
*χ*^2^/sd	0 ≤ *χ*^2^/sd ≤ 3	3 < *χ*^2^/sd ≤ 5
RMSEA	00 ≤ RMSEA ≤ 0.05	0.05 < RMSEA ≤ 0.08
TLI	0.95 ≤ IFI ≤ 1.00	0.90 ≤ IFI < 0.95
NFI	0.95 ≤ NFI ≤ 1.00	0.90 ≤ NFI < 0.95
CFI	0.95 ≤ CFI ≤ 1.00	0.90 ≤ CFI < 0.95
SRMR	0.00 ≤ SRMR ≤ 0.05	0.05 < SRMR ≤ 0.10
GFI	0.95 ≤ GFI ≤ 1.00	0.90 ≤ GFI < 0.95

Byrne (2016), Kline (2016) and Meydan and Şeşen (2011).

### 2.5 Measurements

The PsyCapS and QWLS were used in the study with permission. Information about the validity and reliability of the scales is given below.

#### 2.5.1 PsyCap scale

The PsyCap Scale (PCQ-24) was developed by Luthans et al. (2007a). Erkuş and Afacan Fındıklı (2013) adapted the scale to Turkish. The scale was translated from English to Turkish using the translation-back translation method. This translation was then checked by English Language and Literature faculty members. The questionnaire includes four dimensions (self-efficacy, hope, optimism and resilience) and a total of 24 items for determining PsyCap (Erkuş and Afacan Fındıklı, 2013, pp. 307–308). The self-efficacy dimension was measured by the first six items, the hope dimension by the 7th–12th items, and the resilience dimension by the 13th–18th items. Items and the optimism dimension are measured by items 19–24. The reverse expressions in the original scale have been corrected. As a result of the reliability analysis performed in line with the validity and reliability studies of the scale, the total Cronbach’s Alpha reliability coefficient is 0.89. As a result of the reliability analyses performed for each sub-factor; it was found that the Cronbach’s Alpha reliability coefficient of the self-efficacy factor was 0.90, the hope factor was 0.79, the psychological resilience factor was 0.72 and the optimism factor was 0.68 (Erkuş and Afacan Fındıklı, 2013).

#### 2.5.2 QWLS

The QWLS was developed by Erdem (2008). The scale consists of 43 items. The scale has four items (1–4) in the total living space sub-dimension, seven items (5–11) in the safe and healthy life sub-dimension, six items (12–17) in the employee capacity development sub-dimension, seven items (18–24) in the social responsibility sub-dimension, five items (25–29) in the social integration sub-dimension, seven items (30–36) in the democratic environment sub-dimension and seven items (37–43) in the appropriate and fair wage sub-dimension.

Since the scale does not have exploratory and confirmatory factor analysis, its structural validity was tested with exploratory factor analysis with SPSS 27. Factor analysis was performed to examine the structural validity of the scale formed as a result of the analysis; The KMO coefficient and Barlett Sphericity value of the 43 items that passed the reliability test were found to be 0.928 and 0.000, respectively. As a result of direct oblimin rotation, the items were divided into seven factors and the total variance was found to be 63.64%. The lower limit of the item factor loadings was 0.40 and the difference between two factor loadings of the same item was at least 0.10. Items 11, 12, 13, 14, 15, 27 and 35 that did not meet this criterion were removed from the scale. Exploratory factor analysis was performed on 36 items again to observe the change after the items were removed from the scale.

The general reliability of the scale, KMO value, Barlett Sphericity sig. value was found to be 0.000 and the total variance was found to be 66.71%. The scale was again divided into seven factors. However, the factor names were renamed in accordance with the literature. Cronbach Alpha Coefficient of the 1st factor was found as 0.789, Cronbach Alpha Coefficient of the 2nd factor was found as 0.870, Cronbach Alpha Coefficient of the 3rd factor was found as 0.869, Cronbach Alpha Coefficient of the 4th factor was found as 0.532, Cronbach Alpha Coefficient of the 5th factor was found as 0.813, Cronbach Alpha Coefficient of the 6th was found as 0.904 and Cronbach Alpha Coefficient of the 7th was found as 0.929. The general reliability of the scale was found as 0.937. Table 3 shows the items and factor names, factor loadings, item total correlations, Composite Reliability and Average Variance Extracted values.

**Table 3:** Quality of work life scale factor names, factor loadings and item-total correlations.

Factor name	Items	Factor loadings	Item total correlation	CR	AVE
General wellbeing alpha: 0.789	Item 1	0.753	0.582	0.87	0.62
	Item 2	0.731	0.534		
	Item 3	0.859	0.703		
	Item 4	0.796	0.603		
Working conditions alpha: 0.870	Item 5	0.643	0.534	0.90	0.60
	Item 6	0.784	0.642		
	Item 7	0.849	0.762		
	Item 8	0.826	0.703		
	Item 9	0.771	0.712		
	Item 10	0.740	0.701		
Social responsibility alpha: 0.869	Item 16	0.681	0.670	0.88	0.59
	Item 17	0.768	0.685		
	Item 18	0.884	0.767		
	Item 19	0.833	0.739		
	Item 20	0.650	0.609		
Accountability alpha: 0.532	Item 21	0.867	0.374	0.74	0.59
	Item 22	0.661	0.374		
Social integration alpha: 0.813	Item 23	0.796	0.682	0.82	0.53
	Item 24	0.655	0.593		
	Item 25	0.768	0.699		
	Item 26	0.680	0.561		
Democratic environment alpha: 0.904	Item 28	0.812	0.751	0.92	0.58
	Item 29	0.803	0.689		
	Item 30	0.709	0.681		
	Item 31	0.763	0.750		
	Item 32	0.799	0.650		
	Item 33	0.762	0.713		
	Item 34	0.808	0.749		
	Item 36	0.646	0.598		
Reasonable and fair wage alpha: 0.929	Item 37	0.823	0.746	0.94	0.70
	Item 38	0.851	0.780		
	Item 39	0.769	0.699		
	Item 40	0.874	0.818		
	Item 41	0.845	0.793		
	Item 42	0.870	0.820		
	Item 43	0.826	0.766		

A multiple normality test was conducted to assess the normality distribution of the QWLS. In addition to this, Skewness and Kurtosis values of the distributions were examined. The analysis was performed using data collected from 369 participants. The results indicated that the subscales, wellbeing (Skewness = −0.167, Kurtosis = 0.038), Suitable Fair Fee (Skewness = −0.188, Kurtosis = −0.545), Democratic Environment (Skewness = −0.479, Kurtosis = −0.345), Social Integration (Skewness = −0.388, Kurtosis = −0.002), Accountability (Skewness = 0.126, Kurtosis = −0.040), Social Responsibility (Skewness = −0.368, Kurtosis = −0.296), Work Conditions (Skewness = −0.096, Kurtosis = −0.038), had skewness and kurtosis values within the accepted range −1.5 ile +1.5, as suggested by Tabachnick and Fidell (2013). This confirms that the data distrubition meets the normality assumption.

In order to see the convergent validity of the QWLS, the average variance explained (AVE) values and the composite reliability value (CR) were calculated. As a result of the calculations, the AVE values of the scale were determined as 0.53–0.70; and the CR values were determined as 0.74–0.94. Cronbach’s Alpha is widely used to determine internal consistency. In a data set analysis, the Cronbach Alpha value range; 0–0.49 unacceptable; 0.50–0.59 weak; 0.60–0.69 questionable; 0.70–0.79 acceptable; 0.80–0.89 good; 0.90–1.00 shows that it has an excellent level of value (Sarmento and Costa, 2017).

Factor loadings are expected to be ≥0.45 (Büyüköztürk, 2014); item total correlations are expected to be ≥0.30 (Büyüköztürk, 2014); Cronbach Alpha and composite reliability coefficients are expected to be ≥0.70; and the average variance explained is expected to be ≥0.50 (Hair et al., 2017; Fornell and Larcker, 1981). Nunnally (1967) suggested that values as low as 0.50 are appropriate for exploratory studies.

In addition to this, another perspective assumes that the AVE value above 0.40 and the CR value above 0.70 indicates convergent validity (Shrestha, 2021). Also, the fact that the composite reliability is greater than the average variance extracted (CR > AVE) (Hair et al., 2017) shows that the convergent validity values of the QWLS are sufficient.

**Figure 2:**
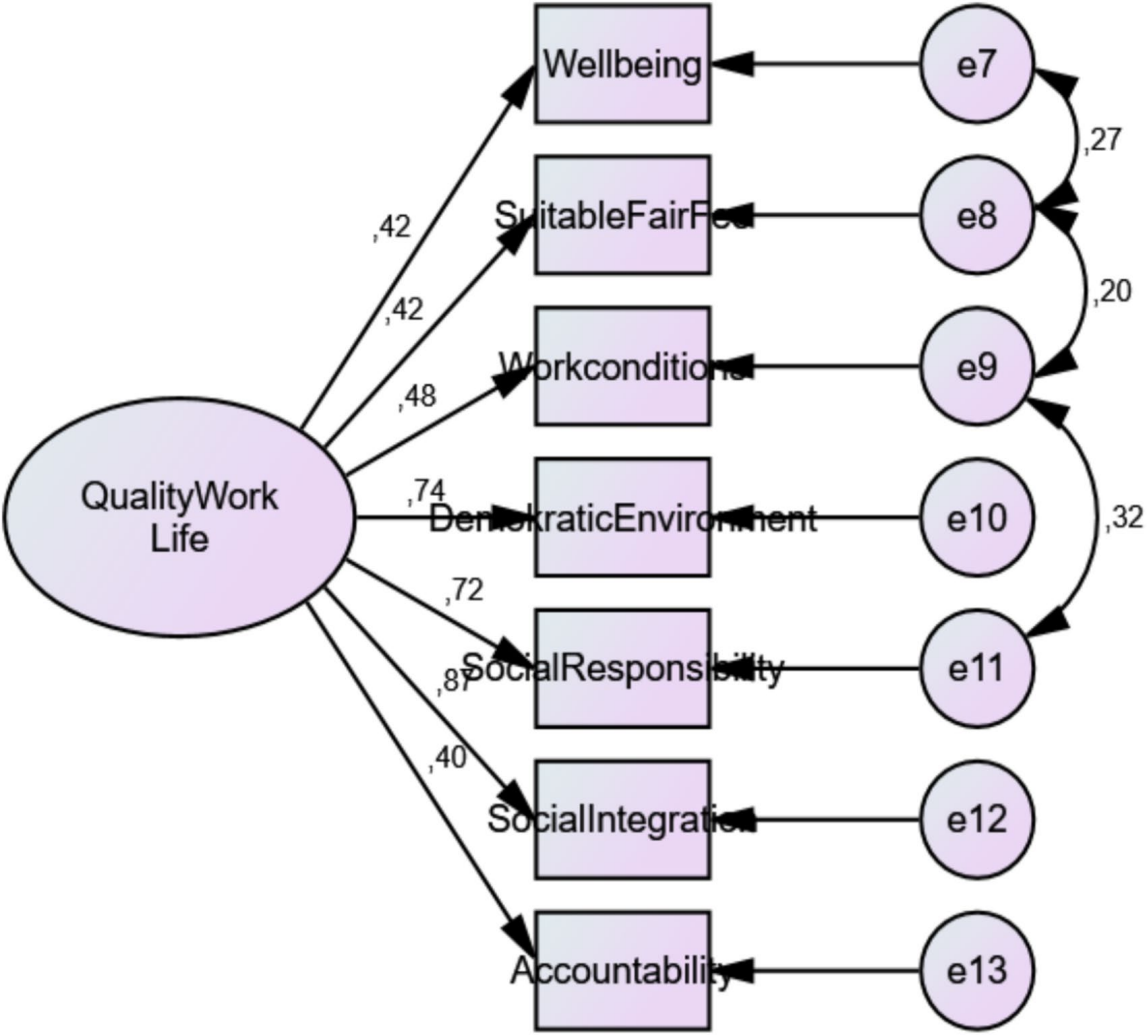
Confirmatory factor analysis of quality of work life.

CFA findings regarding the sub-dimensions of the QWLS prepared within the scope of the study are shown in Figure 2.

The values obtained as a result of the confirmatory factor analysis of the QWLS confirm the factor structure of the scale when the goodness of fit values in Table 2 are taken as basis. In the model fit assessment of structural equation modeling; *χ*^2^/sd, NFI, TLI, CFI, GFI, SRMR and RMSEA fit indices were included. It was determined that the goodness of fit index findings was within the threshold values and that the structure of the QWLS was compatible with the obtained data, and it was seen that the structural validity of the scale was ensured. The *χ*^2^/sd value of the QWLS was found to be 2.25 < 3. Since this result is less than 3, it can be said that the model provided a good fit (Kline, 2016; Meydan and Şeşen, 2011). The QWLS demonstrates the validity of the model in the model fit goodness indices as RMSEA = 0.058 < 0.08 (Acceptable), TLI = 0.96 (Good fit), NFI = 0.96 (Good fit), CFI = 0.98 (Good fit), SRMR = 0.02 (Good fit) and GFI = 0.98 (Good fit).

#### 2.5.3 The scale on emotions

The Positive and Negative Affect Schedule (PANAS) was developed by Watson et al. (1988). The scale was adapted to Turkish by Gençöz (2000). The sub-dimensions of the scale were defined as independent concepts. The scale’s positive and negative emotional dimensions include 10 statements. The statements in the positive emotional dimension are: 1 (interested), 3 (excited), 5 (strong), 9 (enthusiastic, willing, voluntary), 10 (proud), 12 (alert, alert), 14 (inspired, creative), 16 (determined), 17 (attentive) and 19 (active, lively). The statements in the negative emotionality dimension are as follows: 2 (distressed), 4 (unhappy), 6 (guilty), 7 (frightened), 8 (hostile), 11 (nervous), 13 (shy), 15 (nervous, tense), 18 (uneasy) and 20 (afraid). In the study by Watson et al. (1988), the reliability coefficient of the statements related to positive emotions was found to be 0.88, and the reliability coefficient of the statements related to negative emotions was found to be 0.85. In the study of the scale’s adaptation to Turkish by Gençöz (2000), the reliability coefficients were found to be 0.86 for positive emotionality and 0.83 for negative emotionality.

## 3 Results

### 3.1 Descriptive statistics and inter-correlations

Table 4 shows the minimum, maximum, average and standard deviation values according to the sub-areas of the scales.

**Table 4:** Scores obtained from the scales.

Scale	Sub-scale	*N*	Min	Max	*X*	S.D
Psychological capital scale	Self-efficacy	369	18	30	26.27	2.838
	Hope	369	18	30	25.55	2.769
	Optimism	369	15	30	24.01	3.440
	Resilience	369	15	30	24.77	2.970
Emotion scale	Positive emotions	369	20	50	37.85	6.161
	Negative emotions	369	10	31	15.36	4.679
Quality of work life scale	Living space	369	8	20	14.75	2.409
	Safe and healthy life	369	7	35	22.84	5.217
	Development of employee	369	8	30	21.75	4.313
	Capacity	369	9	35	25.00	4.573
	Social responsibility	369	11	25	19.93	3.059
	Social integration	369	16	35	28.73	4.342
	Democratic environment adequate and fair wage	369	7	35	21.29	6.549

Table 5 shows the correlation analysis findings to reveal the relationships between PsyCap, emotions and QWL.

**Table 5:** Correlation values between variables.

Variables	*M*	SD	Psychological capital	Emotions	Quality of work life
Psychological capital	4.19	0.417	1		
Emotions	2.66	0.321	0.331^**^	1	
Quality of work life	3.54	0.498	0.414^**^	0.274^**^	1

** Significant at *p* < 0.01 level.

Table 5 shows that teachers’ perceptions of PsyCap ( $\bar{x}$ = 4.19; SD = 0.417) and QWL ( $\bar{x}$ = 3.54; SD = 0.498) are higher than their perceptions of emotions ( $\bar{x}$ = 2.66; SD = 0.321). Correlation coefficient values are also given in Table 5. Correlation coefficient values; 0.10–0.29 are interpreted as small, 0.30–49 as medium and 0.50–1.00 as high (Cohen, 1988). Accordingly, when Table 5 is examined, it is seen that there is a positive and significant and positive relationship between all variables at the *p* < 0.01 level. It was concluded that there is a positive and moderate relationship between PsyCap and emotions (*r* = −0.331; *p* < 0.01), a positive and moderate relationship between PsyCap and QWL (*r* = 0.414; *p* < 0.01), and a positive and low relationship between emotions and QWL (*r* = −0.274; *p* < 0.01).

### 3.2 Examining the effect of emotions on QWL in the context of PsyCap

#### 3.2.1 Testing measurement models

A confirmatory factor analysis was conducted to test the compliance of the subscales in the PsyCap, emotion and QWL scales to the structure they are in, which will be used in the mediator variable analysis.

**Figure 3:**
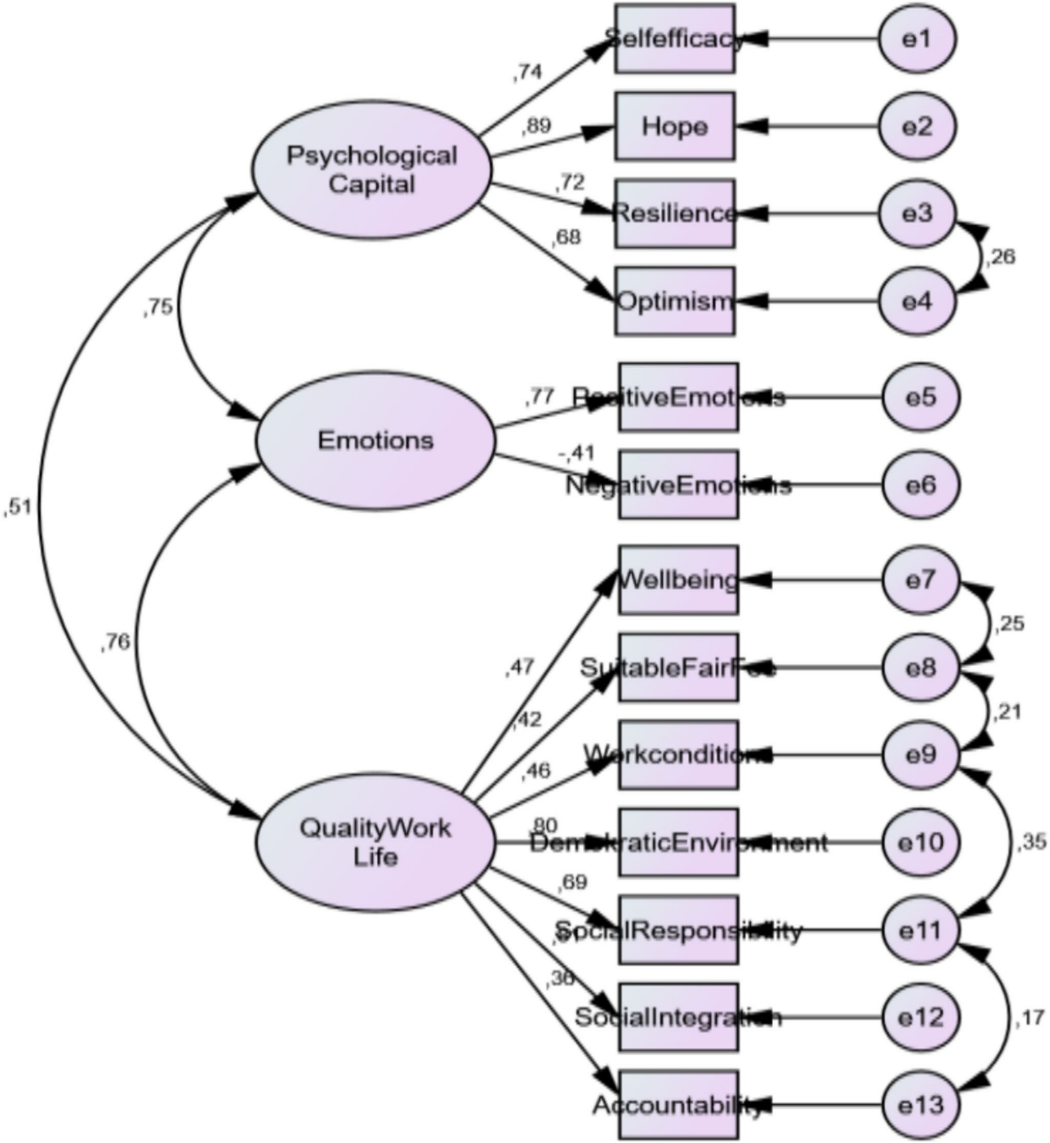
Path diagram of measurement models.

The fit values and results obtained from the Confirmatory Factor Analysis performed are given in Table 2 according to the criteria of good fit and acceptable fit values. According to the principles and criteria of structural equations (Kline, 2016; Kang et al., 2018), the CMIN/DF of the structural equation model in this study was 2.86 < 3, a good fit; RMSEA = 0.071 < 0.08, SRMR = 0.55 < 0.10 and all other fit indices, GFI = 0.935, TLI = 0.920, CFI = 0.942, NFI = 0.914, which are all greater than 0.9, indicating that the data and the model are in good agreement. It can be said that the model-data fit is achieved by evaluating the fit index values from a holistic perspective, together with the t values of the items in the established model being not significant and the errors not being high. The standardized path coefficients for the established model are shown in Figure 3.

**Figure 4:**
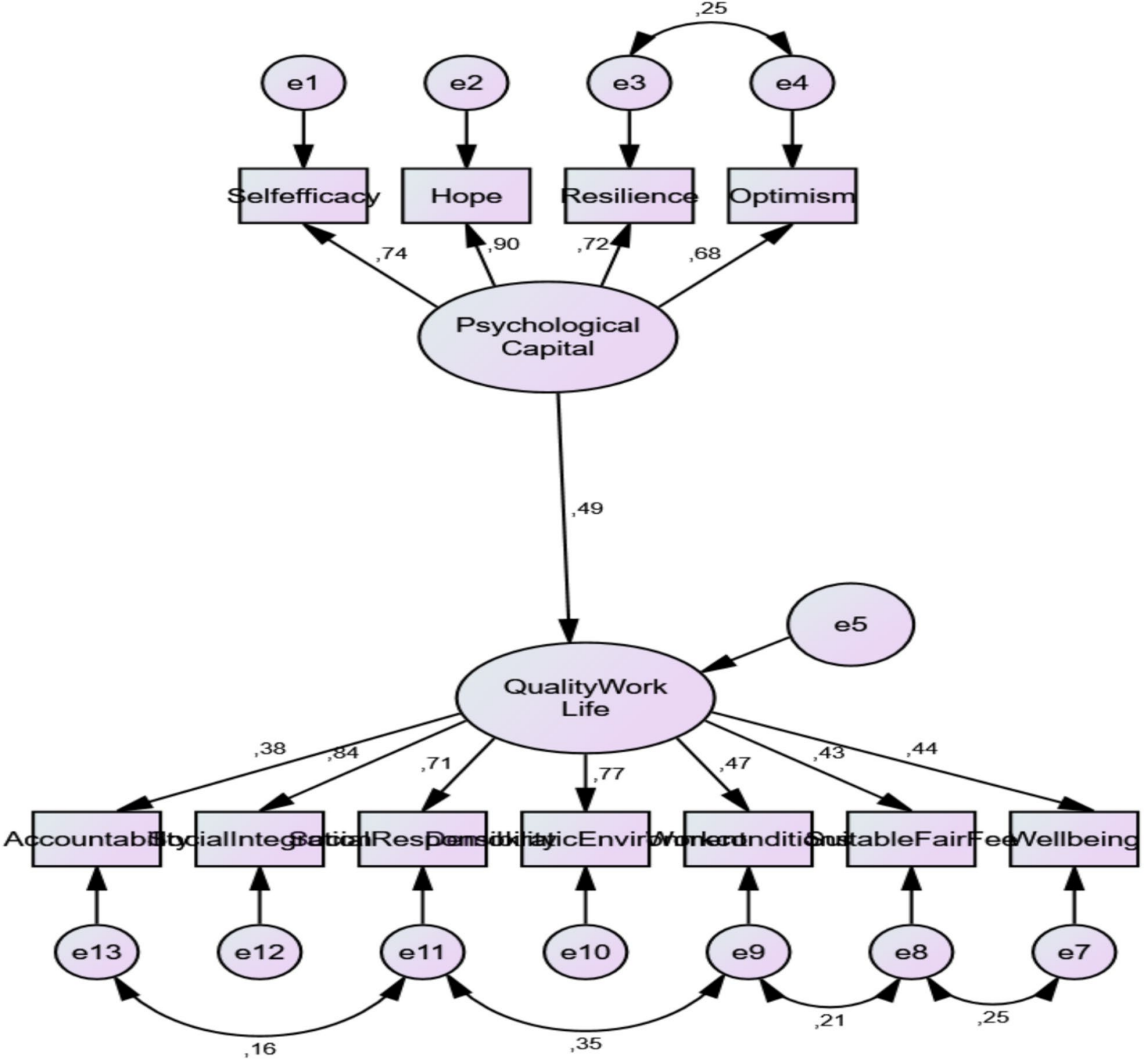
Path model of the effect of psychological capital on quality of work life.

Examining the Relationship Between PsyCap and QWL to test the hypothesis of PsyCap → QWL (Assumption 1), a Structural Equation Model (SEM) was used with PsyCap as an external variable and QWL as an internal variable. The SEM results indicated that PsyCap significantly predicted QWL (*β* = 0.49; *p* < 0.01). The established model and its standardized path coefficients are illustrated in Figure 4.

As seen in Figure 4, only one modification was made in the path analysis. The model fit indices related to the path analysis (*χ*^2^/sd = 3.32; RMSEA = 0.079, NFI = 0.92, TLI = 0.92, CFI = 0.94, GFI = 0.94 and SRMR = 0.059) indicate that the model has an acceptable fit according to the fit criteria in Table 2. The path coefficients obtained as a result of the analysis are given in Table 6.

**Table 6:** Path analysis coefficients related to the prediction of the perception of quality of work life by psychological capital.

Predictor		Predicted	*B*	Standard *β*	*t*	SE	*p*
Psychological capital	→	Quality of work life	0.25	0.49	6.001	0.42	0.000^*^

* Significant at *p* < 0.001 level.

As can be seen in Table 6, PsyCap (*β* = 0.49, *t* = 6.001, *p* < 0.001) was determined to be a positively significant predictor of the perception of QWL. It was determined that PsyCap explained 24% of the variance in the perception of QWL (*R*^2^ = 0.24).

#### 3.2.2 Testing the mediating relationship

When the methods applied to test the mediating effect were examined, it was seen that two different methods were applied. The first of these is the causality approach known as the traditional approach, and the second is the use of bootstrap or resampling methods, which are the contemporary approaches frequently seen in studies recently. In this study, a path analysis based on the bootstrap method was conducted in order to test whether the emotion variable has a mediating role in the relationship between PsyCap and QWL.

The 5,000 resampling option was preferred in the bootstrap analysis. In order to support the research hypotheses in the mediation effect analyses conducted with the bootstrap technique, the values in the 95% confidence interval (CI) obtained as a result of the analysis should not include the value zero (0) (Baron and Kenny, 1986; Hayes, 2009).

**Figure 5:**
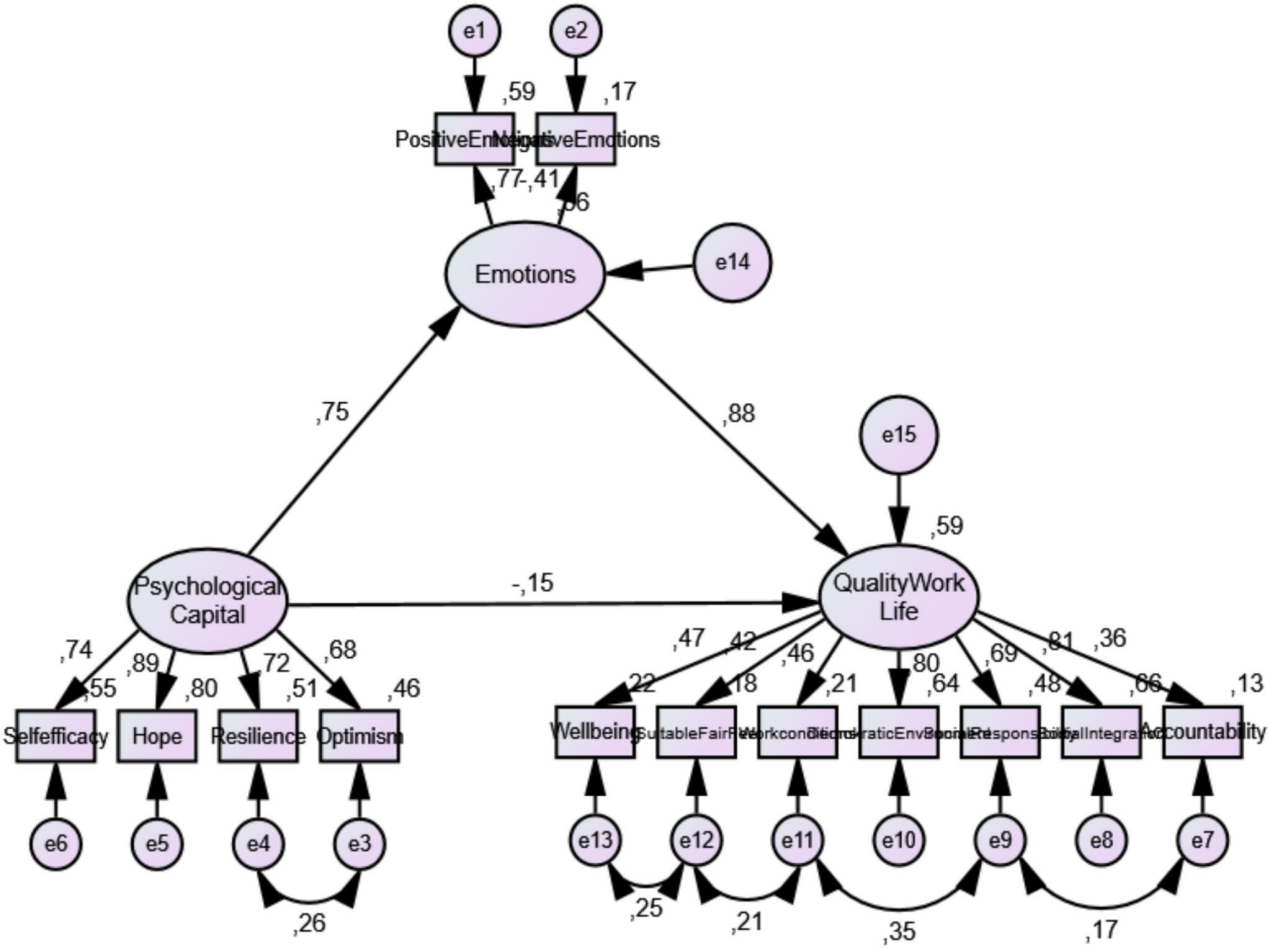
Path diagram of the mediated structural model.

The model mediated by emotions and its standardized path coefficients are shown in Figure 5.

The fit indices obtained as a result of the path analysis are within the acceptable threshold value according to the fit indices in Table 2, indicating that the model is compatible with the data (*X*^2^/sd =2.86; RMSEA = 0.071, NFI = 0.914, TLI = 0.92, GFI = 0.935, CFI = 0.942, and SRMR = 0.055). The path diagram of the model is shown in Figure 5.

According to the results of the mediated structural model analysis presented in Table 7, PsyCap significantly predicted emotions (*β* = 0.75; *p* < 0.01), thus supporting assumption 2. Additionally, the mediating variable, emotions, had a significant effect on the QWL (*β* = 0.88; *p* < 0.01), which supported assumption 3. However, when emotions were included as a mediating variable in the model, the path coefficient from PsyCap to the QWL became non-significant (*β* = −0.15; *p* > 0.05).

**Table 7:** Direct, total and indirect effect SEM analyses.

Direct effect	*B*	*β*	S.E.	*p*	95% CI	
					LLCI	ULCI
PC → E	1.521	0.750	0.151	0.001	0.634	0.874
PC → QWL	−0.37	−0.151	0.050	0.459	−0.932	0.175
E → QWL	0.105	0.876	0.035	0.002	0.523	1.653
Total effect						
PC → QWL	0.123	0.506	0.026	0.001	0.397	0.600
Indirect effect						
PC → E → QWL	0.159	0.657	0.327	0.001	0.353	1.467

All model explained 59% of the variance in quality of work life.

PC, Psychological Capital; QWL, Quality of Work Life; E, Emotions; SE, Standard errors; CI, Confidence interval; LLCI, Lower limits confidence interval; ULCI, Upper limits confidence interval.

Moreover, Table 7 shows that the total effect of PsyCap on the QWL was found to be statistically significant (*β* = 0.251; *p* < 0.001). The bootstrap results indicated that the indirect effect between PsyCap and the QWL was calculated as 0.159, with a 95% confidence interval ranging from 0.353 to 1.467. Since this interval did not include the value 0, the indirect effect was deemed statistically significant. If there is a non-significant relationship between X (PsyCap) and Y (QWL, represented as c′), it suggests a full mediation effect. Conversely, if there is a decrease in the relationship (c′ < c, but c′ is significant), it indicates a partial mediation effect (Baron and Kenny, 1986). In this study, emotions were found to have a full mediation effect since there was no significant relationship between PsyCap and the QWL when the mediator variable was considered. Thus, 0.51 of the total predictive effect of PsyCap on the perception of QWL, 0.66 of the total predictive effect of PsyCap is realized through emotions.

## 4 Discussion

The results of this study compellingly support all four hypotheses, revealing the crucial mediating role of emotions in the relationship between PsyCap and QWL.

Hypothesis 1 was clearly validated, demonstrating that PsyCap profoundly enhances the QWL, as confirmed by the analysis (*β* = 0.49; *R*^2^ = 0.24; *p* < 0.001). These findings resonate with those of Bıyık and Aydoğan (2014), who, in their study of bank employees, established a positive and significant connection between dimensions of PsyCap- such as optimism, resilience, hope, and self-efficacy- and QWL. Their research indicated that these dimensions accounted for an impressive 47% of the variance in QWL. Similarly, Nguyen and Nguyen (2012) corroborated these results in their investigation of PsyCap’s impact on job performance and quality of life among marketers, further strengthening the argument. Additionally, Mortazavi et al. (2012) highlighted the significant influence of PsyCap on QWL and organizational performance in a sample of nurses. Furthermore, in their research on hotel employees, Soybalı and Akerik (2022) concluded that a moderately strong and significant relationship exists between PsyCap and QWL. Notably, this study involving teachers also affirmed that PsyCap plays a vital role in shaping the perception of QWL. The evidence is clear: investing in PsyCap is key to fostering a thriving work environment.

Hypothesis 2 was confirmed through analysis, demonstrating that PsyCap positively and significantly influences emotions (*β* = 1.521; *R*^2^ = 0.563; *p* < 0.001). Avey et al. (2008) found that PsyCap is linked to positive emotions, suggesting that employees with high PsyCap can positively impact their attitudes and behaviors at work through their emotional states. Most research on PsyCap and emotions is related to emotional intelligence (Şener, 2021; Zhang and Xu, 2024; Rodrigues and Junça Silva, 2024) and emotional labor (Tosten and Toprak, 2017; Çelik, 2023). Özdemir and Tarım (2022) discovered that PsyCap positively affects emotional commitment among teachers. Beğenirbaş (2015) reported that PsyCap significantly predicts all sub-dimensions of emotional labor. Similarly, Tosten and Toprak (2017) found that teachers’ PsyCap competencies influence their tendency to exhibit emotional labor behaviors. Gong et al. (2018) also identified a positive relationship between employees’ positive emotions and PsyCap. Huang and Lee (2013) revealed a negative correlation between emotional exhaustion and PsyCap, as well as between a healthy lifestyle and emotional exhaustion.

Hypothesis 3 was confirmed, indicating that emotions positively and significantly affect the perception of QWL (*β* = 0.88; *R*^2^ = 0.563; *p* < 0.001). Elmas and Yıldız (2021) found a positive and moderate relationship between teachers’ perceptions of QWL and positive emotions, while identifying a negative and low-level relationship between negative emotions and perceptions of QWL. Dreer (2021) noted that positive emotions in the workplace play a crucial role in teachers’ job satisfaction. The working conditions of an organization, along with its cultural and psychological structure, can influence productivity. By prioritizing employee wellbeing and fostering a positive perception of the QWL, organizations can enhance this effect. Bilgin (2019) suggested that individuals with positive emotions experience higher job satisfaction and improved job performance. Similarly, Robbins and Judge (2024) asserted that emotional reactions- whether positive or negative- can affect job satisfaction and involvement. People with positive emotions are more likely to perceive their QWL favorably. Events in the workplace can trigger positive or negative emotional responses from employees. Mustafayeva and Ustun (2018) found that increased emotional intelligence correlates with a higher perception of QWL. Titrek (2004) conducted a study with university faculty members and found an interesting contrast. While the faculty generally perceived themselves as competent in using emotional intelligence skills, they still faced significant challenges in several key areas. These included balancing positive and negative emotions, accurately understanding their own feelings, maintaining self-control in stressful situations, sensing opportunities, influencing colleagues, understanding social networks, helping pessimistic coworkers adopt a more optimistic mindset, recognizing different perspectives, interpreting gestures and facial expressions, working effectively within a team, demonstrating leadership, and persuading others.

In Hypothesis 4, the study examined the relationship between PsyCap and QWL, finding that emotions play a full mediating role. The analysis yielded a coefficient (*β*) of −0.159, with an *R*^2^ value of 0.591 and a *p*-value of less than 0.001. According to the Bootstrap results, the indirect effect of PsyCap on QWL was calculated to be 0.159, with a 95% confidence interval ranging from 0.353 to 1.467. Since this interval did not include the value of 0, the indirect effect was considered statistically significant. Emotions can be understood as complex psychological states that are generally intense but short-lived, arising from a person’s interpretation and reaction to specific events or situations. They encompass a variety of feelings, physiological responses, and expressions, and play a crucial role in influencing an individual’s cognition, behavior, and overall wellbeing (Izard, 2009). This study also underscored the importance of emotions as strong mediators in this context.

## 5 Conclusion

This study investigated the mediating role of emotions in teachers’ perceptions of their QWL and PsyCap. Four hypotheses were developed and tested, revealing that emotions fully mediate the relationship between teachers’ PsyCap and their QWL perceptions. The findings demonstrate positive relationships among teachers’ PsyCap, their emotions, and QWL. All four hypotheses were supported, and recommendations for practitioners and academics in the field were provided.

Teaching is a highly respected and valuable profession that deserves recognition and support. It is crucial for teachers to feel valued, as their emotional wellbeing directly impacts their effectiveness in the classroom. By fostering a positive emotional environment, teachers can be empowered to develop constructive approaches to their profession, ensuring sustainability in their growth. Recognizing that teachers’ perceptions of their work life are deeply affected by their emotions, teachers’ mental health of must be prioritized by providing continuous feedback and support. A teacher who is frustrated, unhappy, or stressed- expressing these feelings through raised voices- will struggle to connect with both colleagues and students. This negative energy does not just affect personal interactions; it can lead to unfavorable outcomes throughout the entire school community. Emotions are inherently contagious, influencing everyone they touch, which is why cultivating a positive emotional climate is essential. By prioritizing teacher wellbeing, organizational behavior can be enhanced and ultimately a thriving educational environment for all stakeholders can be created.

To foster a positive emotional climate among teachers, it is crucial to recognize the impact that school principals and vice principals have in shaping teacher experiences. Oppressive attitudes must be replaced with supportive and participatory approaches that empower teachers. While adherence to lesson regulations is important, the emotional states of teachers- whether uplifting or challenging- can drastically influence lesson outcomes. Therefore, school leaders hold a vital responsibility in cultivating a positive organizational culture. The findings inform the development of targeted interventions designed to bolster teachers’ PsyCap and help manage their emotional experiences, leading to an overall enhancement of their QWL. Potential interventions could include professional development programs aimed at fostering resilience, optimism, and emotional regulation among educators (Liu and Du, 2024; Madigan et al., 2023). Professional development programs could include workshops, emotional regulation trainings and mentorship programs fostering emotions of people serving at the educational arena. Teachers especially strengthened with such kind of professional development programs can self-control their emotional regulation skills. Educators equipped with strategies on managing their emotions and how to maintain a positive outlook, can create a more supportive teaching and work environment and can improve teacher-satisfaction and student-outcomes positively. Therefore, implementing professional development programs focused on understanding and managing emotions seems essential. These kinds of programs should also be integrated into teacher training programs, ensuring that emotional intelligence becomes a core component of continuous professional development before and after service. Research by Waber et al. (2021) underscores the importance of emotional awareness during teacher internships, calling for greater emphasis on the emotional dimensions of teaching within teacher education.

This study addresses a significant gap in the literature by exploring how emotions mediate the relationship between teachers’ PsyCap and their QWL. It offers valuable theoretical insights and practical applications aimed at enhancing teacher wellbeing and performance, ultimately benefiting both educators and educational institutions. In conclusion, enhancing the emotional environment within schools will not only benefit teachers but also lead to improved student success, creating a thriving educational ecosystem for all.

## Data Availability

The original contributions presented in the study are included in the article/supplementary material, further inquiries can be directed to the corresponding author.
